# Mapping breast cancer journal publications in conflict settings in the MENA region: a scoping review

**DOI:** 10.3332/ecancer.2020.1129

**Published:** 2020-10-29

**Authors:** Rima A Abdul-Khalek, Ghassan Abu-Sitta, Nassim El Achi, Walaa Kayyal, Ahmad Elamine, Aya Noubani, Marilyne Menassa, Fahad Ahmed, Richard Sullivan, Deborah Mukherji

**Affiliations:** 1 Conflict Medicine Program Global Health Institute, American University of Beirut, Beirut, Lebanon; 2 Institute of Oncology, Hacettepe University, Ankara, Turkey; 3 King’s Health Partners Comprehensive Cancer Centre and Institute of Cancer Policy, King’s College London, London, UK

**Keywords:** breast cancer, research capacity, conflict

## Abstract

**Background:**

Breast cancer is a major cause of cancer-related morbidity and mortality among women in the the Middle East and North Africa (MENA) region. Conflict and political instability in the region may affect medical research output, including that on breast cancer. This scoping review aims to systematically identify and map breast cancer publications across different stages of the cancer care pathway and across conflict-affected countries within the MENA region. The findings of this work will highlight the impact of conflict on cancer research that could be mitigated with the proper contextualised capacity strengthening intervention.

**Methods:**

We followed the PRISMA-Scr methodology. We searched for peer-reviewed publications on topics related to breast cancer in 11 databases: Medline, PubMed, EMBASE, Web of Science, PROQUEST, CINAHL, Global Index Medicus, Arab World Searches Complete, Popline, Scopus and Google Scholar using both controlled vocabulary and keywords. Publication abstracts and full-text versions were screened for duplicates and included in our study based on pre-specified eligibility criteria: focused on breast cancer, related to the specific country of analysis and human or health system studies. We used a structured data extraction form to extract information related to the article, its methodology and the cancer care pathway being studied.

**Results:**

A total of 19,215 citations were retrieved from our search. After removing duplicates, a total of 8,622 articles remained. Title and abstract screening retained 1,613 articles. Publications with first author affiliations to Turkey were consistently the highest across all categories of the cancer care pathway. Trends show an increase in articles from Lebanon, Jordan and Palestine after 2015. Early exploratory and epidemiological studies represented the majority of breast cancer research, followed by policy and implementation research and lastly experimental research. Most research conducted followed an observational study design. Important gaps were identified in the research output related to advanced breast cancer and palliative care (Libya, Syria and Yemen), mental health (Libya), and knowledge and education of breast cancer (Libya and Syria).

**Conclusion:**

This scoping review has identified key areas in breast cancer research that lack significant research activity in conflict MENA settings. These areas, including but are not limited to palliative care, mental health, and education, can be prioritised and developed through regional collaboration and contextualised capacity strengthening initiatives.

## Introduction

According to GLOBOCAN estimates from the International Agency for Research on Cancer (IARC), there were 2.1 million new cases of breast cancer worldwide in 2018 [1], with 626,700 cancer deaths [2], making breast cancer a leading cancer with the second highest incidence after lung cancer [2]. The vast majority of breast cancer cases occur in women, with incidence rates increasing with age. Although breast cancer incidence remains higher in high-income countries employing radiographic screening programs, women in the poorest countries still bear a higher burden of breast cancer mortality [3].

Arab countries comprise the 22 countries of the Arab world, also members of the League of Arab States and are spread geographically from North Africa to Western Asia: Algeria, Egypt, Bahrain, Comoros, Djibouti, Iraq, Jordan, Saudi Arabia, Kuwait, Lebanon, Libya, Mauritania, Morocco, Oman, occupied Palestinian territory, Qatar, Yemen, Somalia, Sudan, Syria, Tunisia and the United Arab Emirates. The Middle East and North Africa (MENA) region includes Arab countries in addition to Turkey and Iran [4]. Compared to Europe and North America, the incidence of breast cancer in Arab countries is significantly lower, but is steadily increasing [5]. Almost half of the patients diagnosed with breast cancer in the Arab region are diagnosed at an age below 50 years, with a median of 49–52 years compared to 63 years in Europe and North America [6]. The tendency for a younger age at presentation has been supported by data from Lebanon [7], Egypt [8] and Yemen [9]. Nevertheless, high-quality registry data are not available in most low- and middle-income countries (LMICs) to estimate the real burden of breast cancer. Reporting of age-standardised incidence rates for breast cancer in countries of the MENA region varies by country and year of reporting and depends on the availability of registry data and the national coverage of registries. For example, reported rates include the following: an average of 91.7 per 100,000 (ASR) in Lebanon for the year 2005–2015 [10] and 33.7 per 100,000 (ASR) in Turkey for 2006 [11]. The IARC reports breast cancer incidence estimates per 100,000 (ASR) for the year 2018 and these estimates can be used for an approximate comparison between countries; for example, estimates show the following figures: Lebanon (97.6), Syria (67.3), Jordan (57.4), Turkey (45.6), Iraq (38.4), Yemen (24.9) and Libya (23.2) [12].

Despite the importance of epidemiological and demographic data to inform policy, there remains a paucity of high-quality information on cancer research across the region that is relevant to policy. Based on the World Health Organisation’s (WHO) priority areas for health research, non-communicable diseases represent a distinct category in which LMICs can contribute to better research [13]. Medical research output varies significantly across the MENA region, with the number of publications per one million people equivalent to one fourth of the worldwide average as reported in a bibliometric analysis on medical research productivity in these countries [14]. The authors in the latter study suggest that political instability, regional conflicts, lack of research infrastructure, ‘brain-drain’ (researchers leaving the area), lack of funding and difficulty of publishing in high-impact journals could all contribute to this paucity of medical research. For breast cancer in particular, a review on breast cancer research activity showed that breast cancer research has been limited in Arab countries compared to non-Arab countries of the Middle East [15]. Many Arab countries in MENA region have witnessed conflicts of different durations. This has added to the complexity of reporting on cancer in general and developing insights from research activity, economic evaluations and patient outcomes research to inform policy-makers.

The aim of this scoping review is therefore to map the landscape of research in breast cancer and assess the productivity in research across different areas of cancer care, with an analysis of trends in publications and research methodologies used by different selected countries in the MENA region specifically affected by conflict. This review followed scoping review methodology in order to broadly explore the different areas of research in breast cancer in the selected countries and was completed by extracting specific data from a large number of research articles. This review is part of the GCRF funded project Research for Health in Conflict MENA (R4HC-MENA) that aims to develop capacities and capabilities in conducting impactful health research in the region with an additional focus on mental health, political economy of health and cancer (R4HC-MENA: developing capability, partnerships and research in the Middle East and North Africa; available at: https://r4hc-mena.org/. Accessed 14 Jan 2020).

## Methods

### Search strategy and study design

A review protocol was developed following the PRISMA-Scr methodology [16] and registered on the Open Science Framework. During July 2018, we searched the following 11 databases for peer-reviewed journal publications on breast cancer: Medline (1946), PubMed (1946), EMBASE (1947), Web of Science (1900), PROQUEST (1905), CINAHL (1937), Global Index Medicus (including IMEMR), Arab World Searches Complete (1972), Popline (1970), Scopus (1823) and Google Scholar. Search strategies were built with the consultation of an experienced medical librarian and using a combination of keywords and controlled vocabulary words that were tailored to the appropriate database to search for articles on breast cancer in specific countries of the MENA region. We used terminologies for breast cancer such as ‘breast neoplasm’, ‘breast cancer’, ‘breast tumour’, ‘breast carcinoma’, ‘metasta*’, ‘adenocarcinoma’, ‘brca’ and ‘hboc’. We used ‘conflict-affected’ to indicate areas that may not be bearing the brunt of violence, but still experience social and political upheaval as a result of conflict, e.g., in the form of an influx of refugees or internally displaced populations in Turkey. MENA region is enduring the burden of multiple and complex emergency situations, where the ongoing armed conflicts are getting more and more protracted. For example, the Syrian, Yemeni and Libyan crises that started in 2011, the earlier Iraqi (since 2003) and Palestinian (since 1948) conflicts along with other emerging conflicts, such as in Lebanon (since 2019), continue to affect the whole region [17]. The region is already facing major rising health challenges like communicable diseases and malnutrition along with the burden of existing communicable and non-communicable diseases including cancer; all of which need to be urgently addressed. The selected MENA countries of analysis recently affected by conflicts and included in our search were Lebanon, Jordan, Iraq, Syria, Palestine, Yemen, Libya and Turkey. Other terminologies used to search for research in these countries were Arab countries, Levant, EMRO, MENA, LMICs and developing countries. All retrieved citations were compiled into one database and duplicates were removed. In addition to the database search, we searched the reference lists of relevant articles on breast cancer in the same countries. No language restrictions were applied.

### Study selection

Teams of two reviewers (RA, NE and AN) independently screened citations retrieved from all the databases according to pre-specified inclusion and exclusion criteria, and in case of uncertainty, a third reviewer was consulted. Full-text articles of included abstracts were reviewed by two reviewers (RA and NE) and inclusion was based on the following criteria: 1) focus is on breast cancer; 2) related to specific country of analysis; and 3) human or health system studies. Articles were excluded if they reported benign conditions, such as granulomatous mastitis, or were conducted on human cell lines or animal studies.

### Charting the data

A data charting form was created, updated when necessary and used to extract information from the included publications. The collected information included the following: title of the publication, name of authors, year of publication, journal of publication, country of affiliation of first author, country of affiliation of senior author, institution of affiliation of first and last author, countries of analysis, funding, funding body name(s), type of publication, study design, type of research (e.g., early exploratory, epidemiological), specific area of research (clinical and biomedical vs. public health), characteristics of the study population (general population, migrants) and place on the cancer care pathway, such as ‘risk factors’, ‘screening and prevention’, ‘knowledge and education’, ‘diagnosis and treatment’, ‘palliative care and metastatic disease’, ‘mental health’ and ‘health system studies’. Health system areas were determined using the WHO’s building blocks for health systems, and included the following categories: service delivery, health workforce, health information systems, essential medicine, financing and leadership governance. The cancer care pathway areas were defined based on specified areas used in guideline development by the Breast Global Health Initiative (BHGI) [18]. We added ‘mental health’ and ‘knowledge and education’ as distinct areas of research, since many studies did not fit into the existing categories. Knowledge and education group of studies included attitudes, behaviour, beliefs, perceptions and related topics, such as perceived barriers to healthcare, as many studies were designed to assess knowledge, attitude and practices collectively, for example. Studies could be mapped into one or more cancer care pathway category. Details on the type of risk factor or treatment studied were also extracted. We considered publications as research papers if they were primary or secondary research papers. Case reports were also included as research papers according to the Web of Science’s definition.

### Statistical analysis

We used STATA 15.1 and R 3.6.0 for statistical analysis. Frequencies and percentages were used for categorical variables. The number of publications was adjusted to the population size using population numbers obtained from the WHO’s worldwide population prospects.

## Results

### Mapping the characteristics of the publications

**Figure 1.**
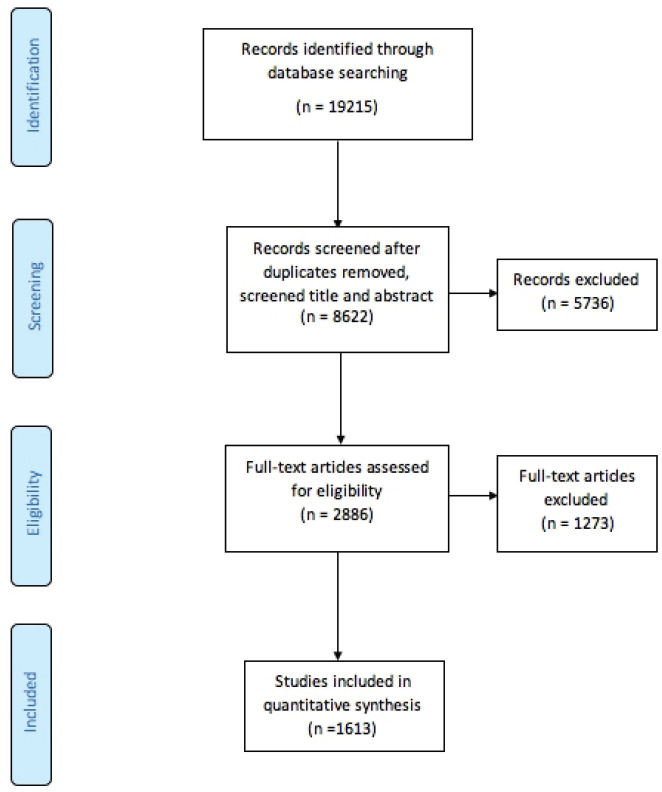
Study flow chart.

From a total of 19,215 publications retrieved (Figure 1), 8,622 were assessed by title and abstract and 2,886 remained for full-text screening. From those, 1,613 were considered eligible for this study and 1,273 were excluded. These excluded publications pertained to two broad categories: general population studies (*n* = 1,167) and studies on migrants or ethnic minority groups (*n* = 106) from the MENA region but residing elsewhere, with the latter being negligible compared to the former. The majority of included publications were original research papers (both primary and secondary research studies; *n* = 1,397). Most of the articles were in English (*n* = 1,572), and the other languages were Turkish (*n* = 34), French (*n* = 4), Arabic (*n* = 2) and Italian (*n* = 1) (Table 1).

**Figure 2.**
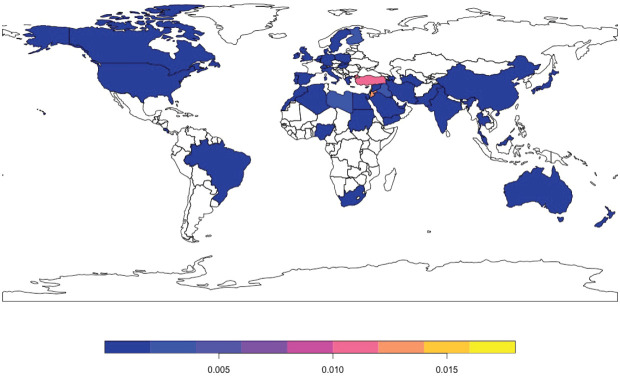
Total number of publications from conflict-affected countries by first author’s country of affiliation.

**Figure 3.**
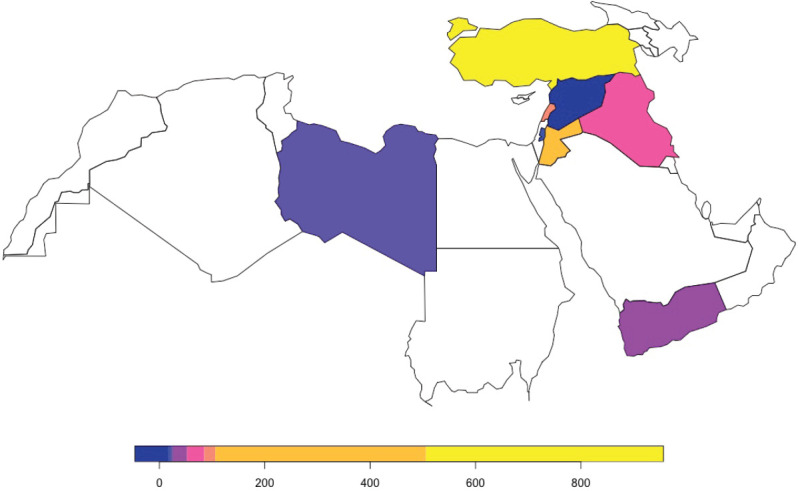
Total number of publications related to conflict-affected countries of the MENA region by first author’s country of affiliation.

When measured by countries of affiliation of first author, Turkey exhibited the highest number of publications, followed by Jordan and Lebanon (Figure 2). Figure 3 shows the geographical distribution of countries of affiliation of first authors of papers related to breast cancer in the MENA population. Results show that authors from all countries across all continents have published on breast cancer in the MENA region.

**Figure 4.**
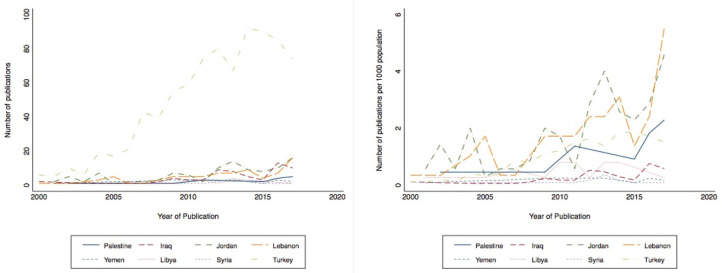
Trends in breast cancer publications by countries of first authors from the MENA region: (a) unadjusted (b) and adjusted for population size.

Trends of publications according to first authors’ countries of affiliation also showed Turkey to be leading in the number of publications on breast cancer (Figure 4). However, after adjusting for population size, Jordan and Lebanon appeared to also have significantly contributed to breast cancer research (Figure 4). Publishing showed an upward trend for Lebanon, Jordan and Palestine, while it showed a decrease in Iraq starting in 2011, followed by Libya, Syria and Yemen. Journals retrieved with the highest number of publications on breast cancer are listed in Table 2.

Most of the publications were published in the *Asian Pacific Journal of Cancer Prevention* (*n* = 161), followed by the *Journal of Clinical Oncology* (*n* = 42), and the *European Journal of Cancer* (*n* = 32). Top institutions of affiliation of primary authors, distributed on the primary countries of analysis of the publication, are presented in Table 3. Most publications were reported from Istanbul University in Turkey, American University of Beirut in Lebanon, The University of Jordan, An-Najah University in Palestine, Baghdad University in Iraq, Damascus University in Syria and University of Aden in Yemen. Similar findings are shown for senior authors from top institutions of affiliation (Table 4).

### Publications across the cancer care pathway

**Figure 5.**
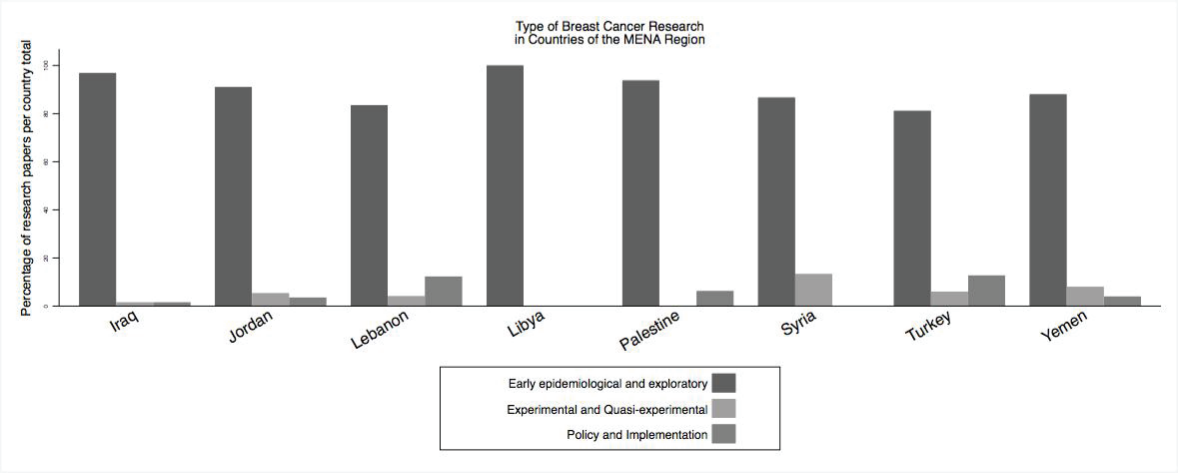
Type of research: early exploratory and epidemiological, trials or health services research.

In terms of research type, most of the research published were early epidemiological and exploratory, followed by policy and implementation then experimental research (Figure 5).

**Figure 6.**
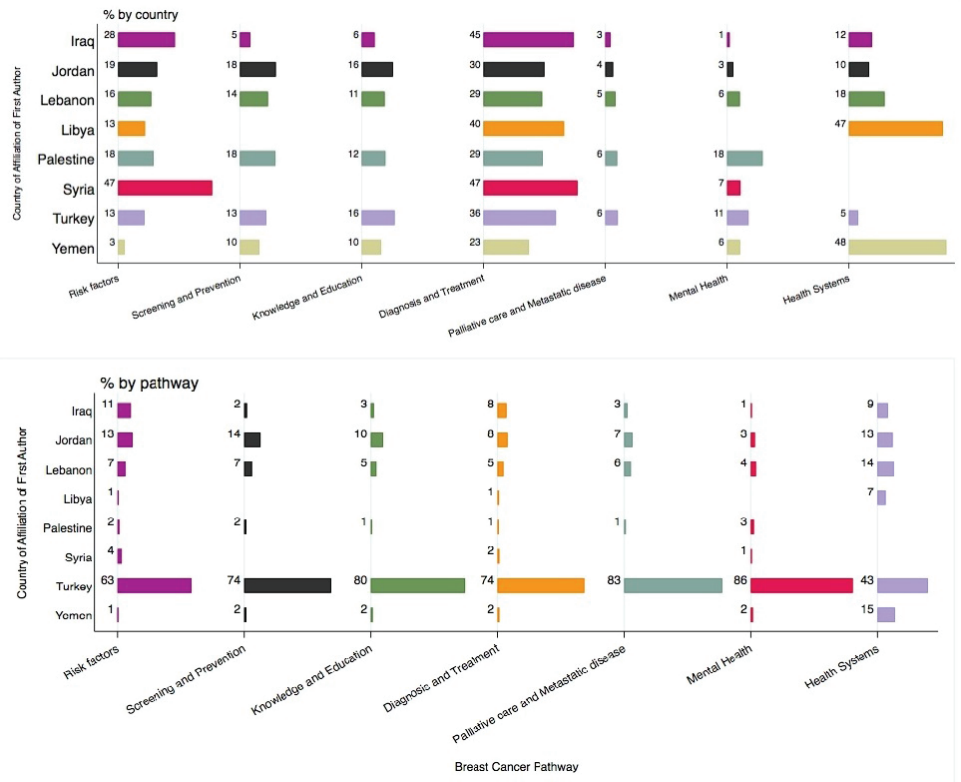
Percentage of publications across the breast cancer care pathway (by country and by pathway).

These publications (both research articles and reviews) were primarily related to diagnosis and treatment of breast cancer (32.44%) and the minority were classified under palliative care and metastatic disease (4.46%) (Figure 6).

When stratified by the country of affiliation of the first author, publications from Turkey were consistently the highest in all cancer care pathway categories: 86% of mental health publications and quality of life, 83% of palliative care and metastatic disease, 80% of knowledge and education publications, 74% of both screening and prevention and of diagnosis and treatment, 63% of risk factor publications and 43% of health system publications.

Research on risk factors was mostly on genetic polymorphisms and mutations associated with breast cancer (*n* = 195). Research on treatment focused on chemotherapy (*n* = 110), followed by surgery (*n* = 93). While risk factors and diagnosis and treatment were the major research areas in breast cancer in the eight countries analysed; gaps were shown in the remaining areas. For example, research on screening, prevention, knowledge and education seem to be non-existent among the research published focusing on Syria and Libya. Mental health is also a gap in Libya, Iraq and Syria, with only (1%) of related publications retrieved. Similarly, palliative care is a gap for Yemen, Syria and Libya. As for health system research, there was a significant gap in this area of research in Palestine and Syria, but was researched in higher proportions in countries other than Turkey (14% for Lebanon and 13% for Jordan). With respect to the study designs adopted, stratification by country of analysis shows that the most common design across all countries was the observational (cross-sectional, case-control, cohort studies, ecological), while other designs were more variable between countries

## Discussion

### Summary of findings

This scoping review of peer-reviewed breast cancer publications from Lebanon, Jordan, Iraq, Syria, Palestine, Yemen, Libya and Turkey showed that Turkey had the highest number of publications. Following Turkey, Lebanon and Jordan appear to have contributed significantly to breast cancer research in the region with upward trends for Lebanon, Jordan and Palestine. As for the conflict-affected countries, like Iraq, Libya, Syria and Yemen, there was a significant decline in publications between 2010 and 2015. The decline could be attributed to the shift in priorities to rapid humanitarian relief rather than to research, or due to the research sanctions imposed on some countries in conflict. In such countries experiencing conflicts, collaborations across disciplines and between partners will be hindered, creating a barrier to research in humanitarian crises, such as conflict, and leading to isolation of research groups [19]. Most of the publications were research articles, and most of the research published followed an observational study design. Important gaps in research outputs from the MENA region were related to advanced breast cancer and palliative care, health system research, knowledge and education on breast cancer and mental health, with significant variations between the countries assessed.

In countries with the lowest number of publications, research seems to specifically tackle risk factors and diagnosis (Libya and Syria). On the contrary, the other assessed countries with a relatively higher number of research papers (Turkey, Jordan, Lebanon and Iraq) covered almost all areas of cancer research. Although Palestine has a lower number of publications than Libya, most areas are researched in comparable proportions, with the exception of health system research. Reasons for variations could be attributed to the lack of expertise and capacity to conduct research in higher impact factor journals, or to limiting knowledge dissemination to local journals of specific institutions or universities which were not included within the scope of this review. Funding and securing fees for publications can be another barrier preventing researchers from publishing research, if conducted [14].

Research on palliative care is particularly important, as it has been shown that there are gaps between recommendations and the practice of palliative care elsewhere, this being mediated by the healthcare financing mechanism [20]. Further research on palliative care in the Arab region can explore this practice, especially in Libya, Syria and Yemen. Initiatives for advancing palliative care research have been implemented in Lebanon with the establishment of research committees in the National Task Force on Pain relief and Palliative care [21].

Our results show that there is a need to strengthen the capacity building in specific areas such as conducting research on health system policy, as improving survival in cancer needs to consider the system that delivers cancer care and not only risk factors for the disease. Based on our findings, there is an urgent need for such an initiative to be implemented specifically in Palestine and Syria, as the other countries have contributed in acceptable proportions to this research area. This is in line with previous recommendations by BHGI of implementation science and cost-effectiveness studies that can guide the establishment of programs for early detection of breast cancer [22]. Implementation research, which focuses on the knowledge of how a healthcare system functions in terms of cancer detection, diagnosis and treatment, is also required in order to ensure the uptake of scientific and clinical research findings and to detect improvements in cancer outcomes [18]. Similarly, health systems and policy research evidence needed for health policy-making in Mediterranean countries is facilitated by the improved methods for health systems and policy research and by building the capacity of policy-makers in assessing the quality, cost-effectiveness of evidence and its applicability [23]. The use of real-world evidence to inform health decision-making in healthcare decision-making is still limited compared to other countries but is expected to improve over the next decade alas the proper capacity strengthening interventions are implemented [24]. In addition, future studies that look into the type of qualitative studies, mapped into the exploratory type of studies in our study and that identify gaps in social, cultural, economic and political barriers to treatment of breast cancer are needed.

### Implications for capacity building

Although medical research in Arab countries was estimated to be lower in quantity compared to the rest of the world for the time period 2007–2016 (189 versus 695 per one million people), the rate of increase was higher for Arab countries in comparison to global rates over this same time period [14]. Therefore, capacity building of health professionals in a region where research is improving in terms of quantity has the potential, if properly addressed, to improve the quality of evidence generated as well as the quality of medical services provided.

However, improving the quality of medical research through capacity building will be more complex than increasing the rate of publications. Evidence from a systematic review, for example, shows that the quality of breast cancer economic research in LMICs is poor [25]. It is important to identify factors that can improve research quality and incorporate them into capacity building initiatives. Thus, filling research gaps in breast cancer should be carried out through robust research that is usable for evidence generation and for policy-making.

Implementation of capacity building initiatives will require that stakeholders be aware of the importance of research capacity building, whether they include strategic entities or individual healthcare providers. Stakeholder interviews exploring breast cancer control strategies in different regions of Asia, Latin America and the Middle East have identified capacity building in research and development of research infrastructure, amongst others, as important strategies for breast cancer control [3]. Similarly, training in clinical research among nurses has been indicated as an important strategy in research capacity building [26], and capacity for clinical research in the Middle East [27]. Limited evidence is available on the perception of the importance of research quality in capacity building studies from the MENA region. Approaches to increase research quality in other fields such as orthopaedic research in LMICs identified academic collaboration, defined as the inclusion of LMIC and non-LMIC investigator, to be associated with higher levels of evidence and thus higher quality studies [28]. In cancer treatment trials, international and national academic collaboration is supported by a way to improve cancer therapy and patient outcomes [29, 30].

### Implications for research context and practice

Lower mortality rates in high-income countries have been attributed to investments in research and improvements in early detection [22]. Thus, integrating research into health systems in developing countries has become necessary to improve health [31]. However, recommendations for more impactful research and for improving the capacity to conduct research necessitate a better understanding of the current state of affairs in research.

The limited amount of reliable data and real-world data in the Middle East contributes to the data poverty in the region. Conducting research in the Middle East faces problems such as data availability and quality when compared to more developed countries [32]. Increasing research in certain areas will need to take into consideration the general challenges for conducting research in times of conflict; these challenges include, but are not limited to, access to information and respondents, and problems of generalisation of findings due to unrepresentative samples [32]. Some of the gaps identified in this review, including mental health research and research data generation, also face issues related to social stigma and fear from social exclusion that arise from cultural norms in some societies.

The difficulty in accessing and generating data is complicated by the political instability in times of war and conflict that may prevent researchers from using some research methodologies, and so different methodologies have been proposed to conduct research in conflict settings [33]. Some related approaches to improving research methods and that address data quality are suggested by Hamadeh *et al* [34] in a review on cancer research in Arab countries. The authors propose more high-quality research evidence on cancer from the Arab world, while others advocate for better resource allocation and utilisation to minimise inequalities in biomedical research productivity. For breast cancer in particular, there is limited evidence on the breadth of the research used to describe breast cancer in the conflict settings of the MENA region, and the amount of training health professionals are provided with to analyse and report data on breast cancer. In Iraq, an initiative for improving data collection on clinicopathological characteristics was developed to support cancer research, this research being regarded as a basic pillar in the cancer control strategies [35]. Additional research endeavours tailored to the identified gaps are recommended in health research areas of mental health, palliative care and education. Exploring the types of barriers in qualitative exploratory studies is also necessary to complement strategies of health research capacity building in cancer.

## Limitations

The limitations of this study, as with other reviews, are related to the methodology of conducting scoping reviews. Our study coverage was complete for articles in international databases; however, we may have missed some articles not indexed by any of the databases, such as articles published by local universities. It is also possible that we did not capture articles by authors from the selected countries of interest due to the affiliations and content of the papers not being amenable to data capture using our methods. We have limited this scoping review to journal article publications for comparability purposes; however, a future grey literature review can complement the finding of this review. Future research can also explore the effect of additional factors on breast cancer research, such as economic development and investment in research capacity building.

## Conclusion

This review is the first to assess the trends of cancer research in conflict-affected countries in the MENA region. It has shown huge disparities between the countries assessed that can be attributed to a wide range of limitations, like funding, facilities and infrastructure, local research capacities and impermissive policies along with the nature of conflict that deprioritises research in comparison to rapid humanitarian relief. The review highlighted the strengths and weaknesses of each of the countries assessed in the different research areas along the breast cancer pathway. The review has also identified key research areas and regional academic hubs that can be the focus for research collaboration and capacity building activities in the region.

## List of Abbreviations

IARC: International Agency for Research on Cancer
LMICs: Low- and Middle-Income Countries
MENA: Middle East and North Africa
WHO: World Health Organisation

## Conflict of interest

All authors have no conflicts of interest.

## Authors’ contributions

RA, RS and GA designed the scoping review. RA, NS and AN screened the retrieved articles. RA, AE and WK extracted data from full-text articles. RA analysed the data and wrote the first draft. All authors reviewed and commented on the final draft.

## Funding

This publication is funded through the UK Research and Innovation GCRF Research for Health in Conflict (R4HC-MENA); developing capability, partnerships and research in the Middle and Near East (MENA) grant number ES/P010962/1.

## Figures and Tables

**Table 1.** Type of publications.

Type	N (%)
Research paper	1397 (86.60)
Abstract	135 (8.36)
Letter to editor; Opinion piece; commentary; editorial	49 (3.03)
Report	13 (0.80)
News	7 (0.43)
Other	12 (0.74)
Total	1613

**Table 2.** Top journals with more than 20 publications on breast cancer.

Journal	Frequency	Percentage
**Asian Pacific Journal of Cancer Prevention**	161	9.98
**Journal of Clinical Oncology**	42	2.60
**European Journal of Cancer**	32	1.98
**Diagnostic and Interventional Radiology**	29	1.80
**Saudi Medical Journal**	29	1.80
**Cancer Nursing**	24	1.49
**Journal of Balkan Union of Oncology**	27	1.67
**European Journal of Oncology Nursing**	21	1.31
**Journal of Cancer Education**	26	1.61
**European Journal of Breast Health**	17	1.05
**Eastern Mediterranean Health Journal**	16	0.99

**Table 3.** Top institutions of affiliation of primary authors in publications from countries of the MENA region (*N* = 1,613).

Country	Institution Name	N
**Turkey (*N* = 895)**	Istanbul University Ege University Hacettepe University Dokuz Eylul University Gazi University Uludag University Maramara University Onodokuz Mayis University Akdeniz University	95 51 45 36 29 26 25 23 17
**Lebanon (*N* = 94)**	American University of Beirut Medical Centre American University of Beirut Saint Joseph University Hotel Dieu de France Lebanese American University	42 21 15 5 1
**Jordan (*N* = 118)**	The University of Jordan Jordan University of Science and Technology Hashemite University King Hussein Cancer Centre Mutah University Zarqa University	24 19 17 10 6 5
**Palestine (*N* = 19)**	An-Najah National University Bethlehem University Al-Quds University Islamic University of Gaza Birzeit University Al-Aqsa University	5 3 3 3 1 1
**Iraq (*N* =76)**	Baghdad University Kufa University University of Babylon University of Basrah University of Al-Qadisiyah Al-Nahrain University	15 8 7 6 5 3
**Syria (*N* = 16)**	Tishreen University Damascus University University of Aleppo Bairouni University Atomic Energy Commission of Syria Damascus Cancer Centre	3 3 1 1 1 1
**Yemen (*N* = 30)**	University of Aden Hadhramout University Sanaa’ University Management and Science University	10 8 5 3
**Libya (*N* = 22)**	Tripoli Medical Centre Benghazi University Garyounis University Qariunis University	6 4 2 2

**Table 4.** Top institutions of affiliation of senior authors in publications from countries of the MENA region.

Country	Institution N	N
Turkey (*N* = 833)	Istanbul University Hacettepe University Ege University Dokuz Eylul University Maramara University Gazi University Uludag University Onodokuz Mayis University Ankara University	94 50 47 39 28 27 24 23 18
Lebanon (*N* = 86)	American University of Beirut Medical Centre American University of Beirut Saint Joseph University Hotel Dieu de France Ministry of Public Health	38 17 8 8 5
Jordan (*N* = 92)	The University of Jordan Jordan University of Science and Technology Hashemite University King Hussein Cancer Centre King Hussein Medical Centre Ministry of Health	20 14 11 11 5 4
Palestine (*N* = 18)	Bethlehem University An-Najah National University Al-Quds University Islamic University of Gaza Birzeit University Ministry of Health	2 4 3 3 2 2
Iraq (*N* = 61)	Kufa University University of Baghdad University of Basrah University of Al-Qadisiyah University of Babylon Al-Nahrain University	7 8 5 4 3 3
Syria (*N* = 12)	Univesity of Aleppo Damascus University University of Aleppo Bairouni University Tishreen University	4 3 1 1 1
Yemen (*N* = 22)	Hadhramout University Sanaa’ University University of Aden Management and Science University	5 4 4 3
Libya (*N* = 16)	Tripoli Medical Centre Benghazi University Qariunis University	4 4 2
